# Cardiac Tamponade-Associated Dense Deposit Disease: A Case Report and Review of the Literature

**DOI:** 10.7759/cureus.29280

**Published:** 2022-09-18

**Authors:** Saeed M Al Zabali, Aljawharah K Rubaihan, Madawi F Alnetaifat, Omer Algonaid, Milad El-Segaier

**Affiliations:** 1 Pediatric Nephrology, King Fahad Medical City, Riyadh, SAU; 2 College of Medicine, AlMaarefa University, Riyadh, SAU; 3 College of Medicine, King Saud Bin Abdulaziz University for Health Sciences, Riyadh, SAU; 4 King Salman Heart Center, King Fahad Medical City, Riyadh, SAU

**Keywords:** cardiomegaly, pericardiocentesis, cardiac tamponade, dense deposit disease, pericardial effusion

## Abstract

Pericardial effusion is an abnormal accumulation of fluid in the pericardial cavity. It can be associated with various cardiac and non-cardiac disorders. Dense deposit disease (DDD) is a rare kidney disease caused by uncontrolled activation of the alternative complement pathway. We are reporting a seven-year-old male child who was diagnosed to have DDD approved by renal biopsy and presented with shortness of breath, cough, and fever. Chest X-ray displayed cardiomegaly. Thereafter, echocardiography showed massive pericardial effusion and left ventricle compression with a risk for cardiac tamponade. He subsequently underwent pericardiocentesis with the removal of 450 ml of pericardial fluid. The patient's edema was not correlated with the described amount of drained pericardial fluid. To the best of our knowledge, this is the first reported case of significant pericardial effusion carrying the risk of cardiac tamponade associated with DDD. With this report, we would like to highlight the importance of cardiac assessment in patients with DDD, in particular those with nephrotic range proteinuria who present with cardiac symptoms and cardiomegaly.

## Introduction

C3 glomerulopathy (C3G) is a form of glomerulonephritis that results from abnormal regulation of the alternative complement pathway leading to C3 deposition in glomerular capillaries. C3G may be categorized into dense deposit disease (DDD) and C3 glomerulonephritis (C3GN) [1-3]. It is ultra-rare with an incidence of approximately one per million per year [2]. C3GN is driven by genetic and/or acquired defects, and dysregulation mostly occurs at the level of the C3 convertase of the alternative pathway in the fluid phase [2]. The presentation is usually a slowly progressive disease with hematuria and non-nephrotic proteinuria, but nephrotic syndrome and more severe presentations have been described [4]. About 50% of patients with DDD progressed to end-stage renal disease (ESRD) within 10 years [5,6]. Cardiovascular complications are a leading cause of death in ESRD in pediatric and adult patients. The pericardial cavity is the potential space between the visceral and parietal components of the pericardium, which is normally lubricated by a very small amount of serous fluid. An abnormal increase in fluid volume leads to pericardial effusion, which could be a result of different etiology [7,8]. Pericardial effusion in renal diseases is usually caused by continuous volume overload as a result of salt and water retention or secondary to hypoalbuminemia leading to shifting of fluid from intravascular into the interstitial compartment [8,9]. Patients with ESRD, in particular, are more likely to develop chronic pericardial effusion due to continuous volume overload [9]. Pericardial effusion and tamponade are extremely rare but serious complications of nephrotic syndrome [10]. When pericardial effusion generates pericardial tamponade, the patient usually develops dyspnea, tachycardia, jugular venous distension, and pulsus paradoxus, and these symptoms lead to hypotension and shock [11].

## Case presentation

A previously healthy seven-year-old boy presented with mild facial puffiness, red-colored urine, and decreased urine output preceded two days by an upper respiratory tract infection. He was edematous with extensive periorbital puffiness, bilateral pitting peripheral edema, and ascites. He presented with a picture of rapidly progressive glomerulonephritis (RPGN). The laboratory findings upon initial presentation are summarized in Table 1.

**Table 1:** Laboratory findings upon initial presentation and during the presentation of cardiac tamponade WBC: white blood cells; RBC: red blood cells; ASO: antistreptolysin O; ANA: antinuclear antibody; ANCA: antineutrophil cytoplasmic antibodies; anti-GBM: anti-glomerular basement membrane; CFH: complement factor H; CFB: complement factor B; CFI: complement factor I; MAC: membrane attack complex (C5b-C9); LDH: lactate dehydrogenase; PCR MTB DNA complex: *Mycobacterium tuberculosis* complex DNA.

Laboratory tests	During initial presentation	One month later, during the presentation with cardiac tamponade
Hemoglobin, g/dl	8.8	9.8
WBC, 10^9^/L	5.3	13.1
Platelets, 10^9^/L	394	630
Creatinine, umol/L	304	69
Urea, mmol/L	19.4	3.5
Albumin, g/L	25	26
Sodium, mmol/L	138 mmol/L	140
Potassium, mmol/L	4 mmol/L	4
Bicarbonate, mmol/L	17	20
C3, g/L	0.1 (0.9-1.8)	0.4 (0.9-1.8)
C4, g/L	0.23 (0.1-0.4)	0.3 (0.1-0.4)
ASO titer, ANA, ANCA, anti-GBM	Negative	Not repeated
CFH	20.1 (23.6-43.1 mg/dl)	Not repeated
CFB	8.1 (15.2-42.3 mg/dl)	Not repeated
CFI	65 (29.3-58.5 mg/l)	Not repeated
MAC	479 (<251 ng/ml)	Not repeated
24-hour urine protein, g/day	8.4	3.4 g/day
Genetic testing	Negative	Not repeated
Pericardial fluid analysis		
Albumin, g/L	NA	5.5
LDH, U/l	NA	53
Glucose, mmol/L	NA	6.3
Count	NA	10/cumm
WBC count	NA	1/cumm
PCR MTB DNA complex	NA	Target not detected
Mycobacterium examination	NA	Negative
Fungal examination	NA	Negative

**Figure 1:**
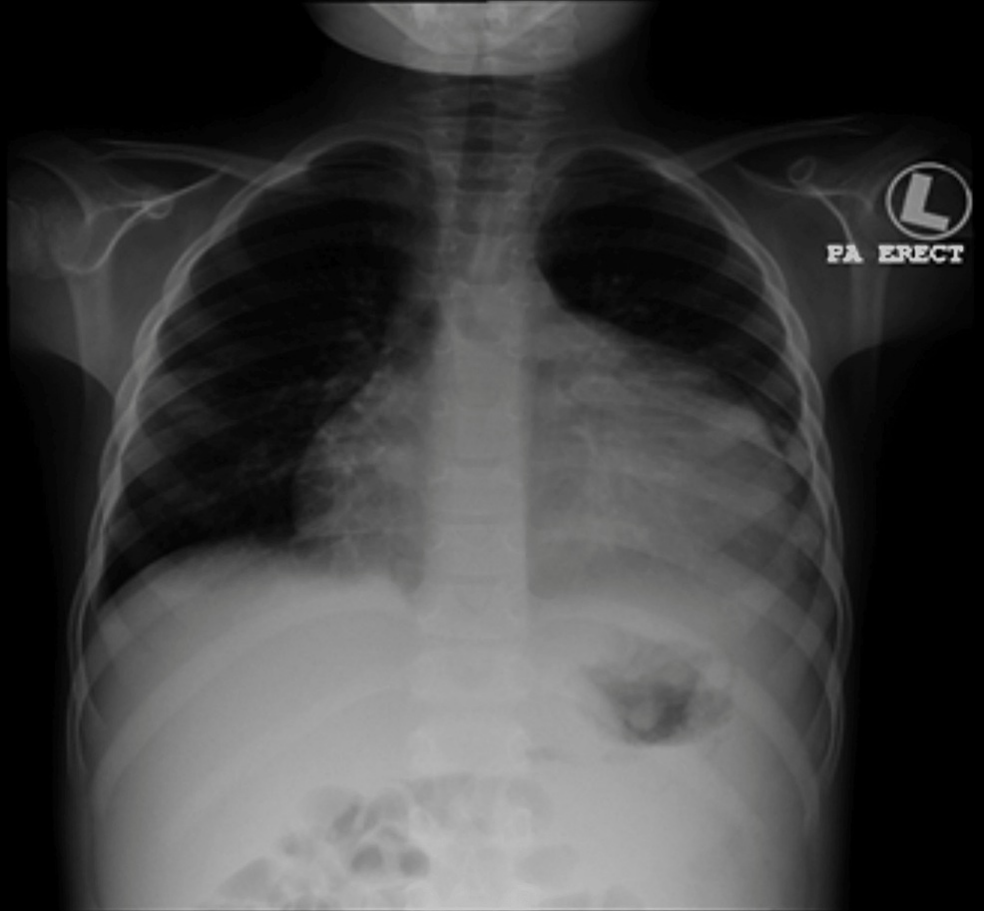
Chest X-ray showing cardiomegaly

**Figure 2:**
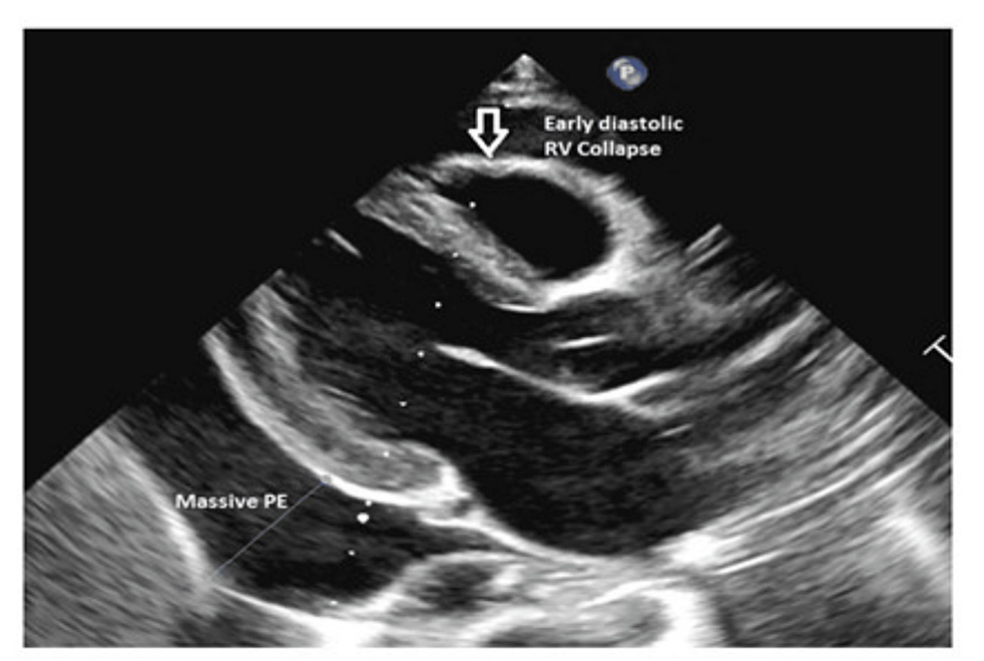
Initial echocardiography showing massive pericardial effusion and early diastolic right ventricular collapse PE: pericardial effusion; RV: right ventricular.

**Figure 3:**
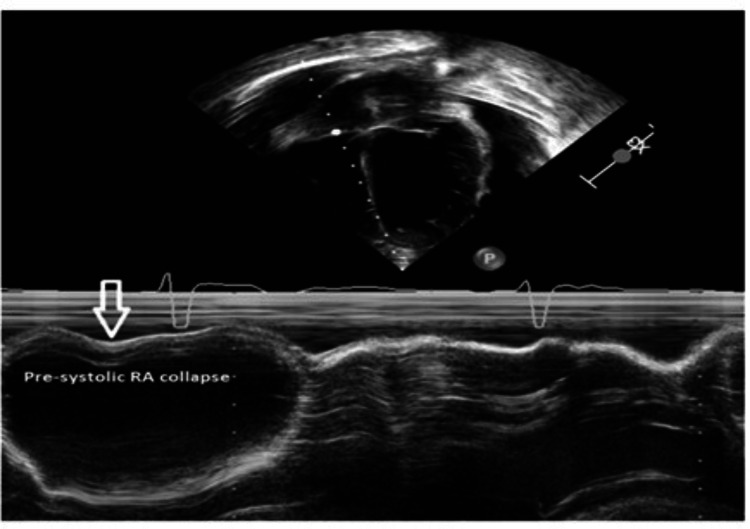
Initial echocardiography showing the pre-systolic right atrial collapse RA: right atrial.

**Figure 4:**
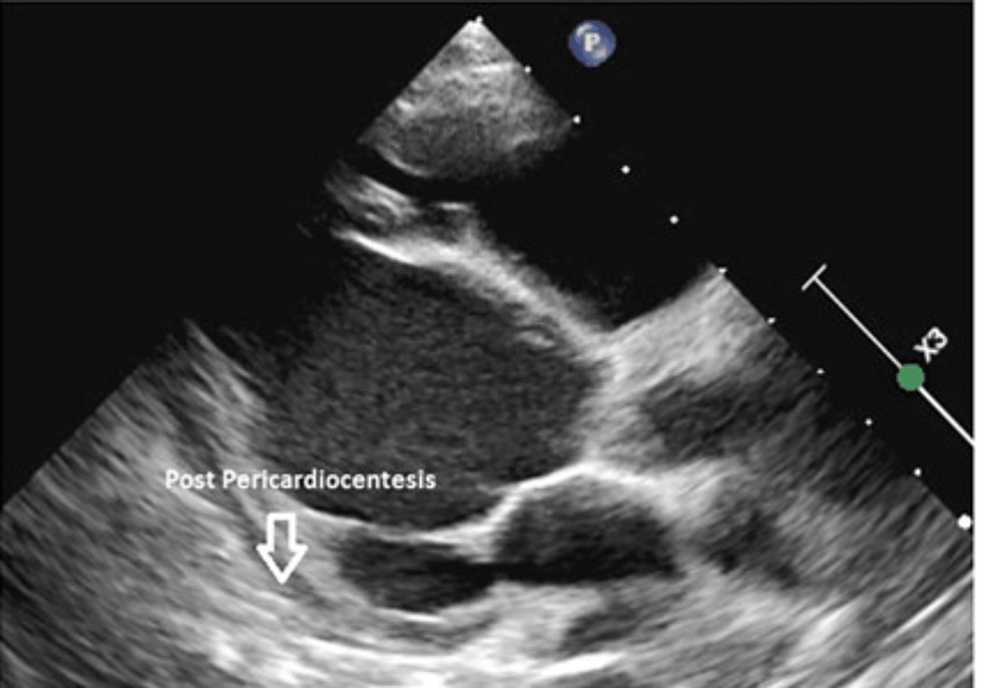
Immediate post-pericardiocentesis showing no pericardial collection

IV methylprednisolone pulse therapy was commenced for five doses daily. Initial echocardiography showed normal cardiac function. A kidney ultrasound revealed echogenic parenchyma with impairment corticomedullary differentiation. His creatinine improved slowly after pulse therapy; edema was treated with albumin and Lasix infusions. Additionally, he had high blood pressure that required multiple antihypertension medications to control and was treated with amlodipine, lisinopril, and atenolol. Renal biopsy revealed DDD. Eculizumab was given according to the dosing regimen established for the atypical hemolytic uremic syndrome, preceded by a meningococcal vaccine. After eculizumab, blood pressure, creatinine, and proteinuria were improved. Mycophenolate mofetil was also prescribed. One month later, he presented with shortness of breath, edema, low-grade fever, and cough. His pulse rate was 102 beats/minute, blood pressure was 114/72 mmHg, respiratory rate was 26/minute, and weight was 24.3 kg. A chest X-ray showed huge cardiomegaly (Figure 1). Echocardiography showed massive pericardial effusion (Figures 2-4).

**Figure 5:**
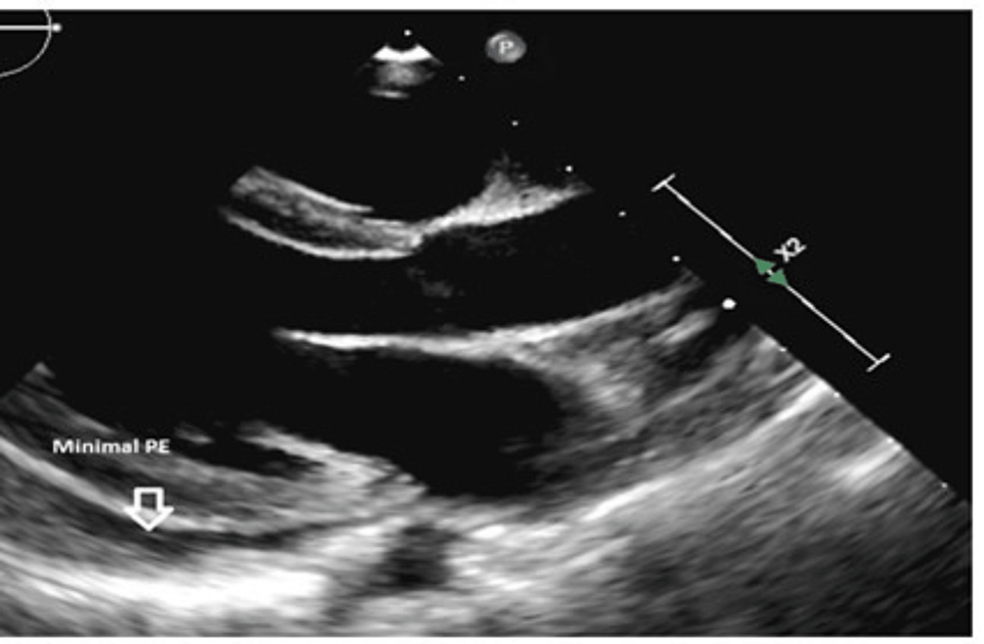
Two weeks post pericardiocentesis showing minimal pericardial recollection PE: pericardial effusion.

His clinical parameters and echocardiography findings indicated a high risk for cardiac tamponade; thus, an urgent pericardiocentesis was performed, where 450 ml of serous fluid was drained. Laboratory testing including pericardial fluid analysis is shown in Table 1. A repeated echocardiography two weeks later showed minimal effusion (Figure 5). Up to the present, he is on regular eculizumab therapy.

## Discussion

Patients with DDD can be presented with asymptomatic microhematuria and/or proteinuria to severe disease with nephritic or nephrotic syndrome and renal impairment [12]. Nephrotic syndrome has been reported up to 38-43% in DDD [12]. Acute cardiac tamponade is a life-threatening condition, caused by pressure on the heart from fluid in the pericardial space that causes a severe decrease in ventricles diastolic filling. Thus, it requires a preemptive diagnosis and treatment [11,13]. The causes of pericardial effusion inducing pericardial tamponade are heart surgery, trauma, chest radiation, tumor, chest radiation, hypothyroidism, ESRD, invasive cardiac intervention, autoimmune disease, and acute inflammatory pericarditis [14]. Pericardial effusion can occur in idiopathic nephrotic syndrome and systemic lupus erythematosus (SLE) [15,8]. Pericardial tamponade is an extremely rare but serious complication of nephrotic syndrome with only a few documented cases in the literature [8,10,16,17]. Table 2 summarizes some cases reported for nephrotic syndrome patients with pericardial tamponade.

**Table 2:** Reported cases of nephrotic syndrome with pericardial tamponade SRNS: steroid-resistant nephrotic syndrome; FSGS: focal segmental glomerulosclerosis; DDD: dense deposit disease; WBC: white blood cells; ESR: erythrocyte sedimentation rate; PCR: polymerase chain reaction; LDH: lactate dehydrogenase.

Case	Deepti Suri et al. (2009) [17]	Aslı Kavaz et al. (2011) [10]	Sunita Namdev et al. (2013) [16]	Present report
Age (Y)	4	8	11	7
Sex	F	F	M	M
Country	India	Turkey	India	Saudi Arabia
Diagnosis	SRNS (FSGS), chylopericardial tamponade, superior vena cava thrombosis	SRNS (FSGS), pericardial tamponade	SRNS (FSGS), pericardial tamponade	DDD, pericardial tamponade
Examination	Anasarca, tachycardia, tachypnea, pulsus paradoxus, decreased urine output	Edema, pallor, blurred consciousness, tachycardia, hypotension	Edema, hypertension tachypnea, and pulsus paradoxus. Heart sounds were muffled	Edema, shortness of breath
Laboratory findings	Hemoglobin: 9.1 g/dl; WBC: 10.25 × 103 cells/μl with 78% polymorphonuclear cells, 19% lymphocytes; platelet count: 665 × 103 cells/μl; ESR: 60 mm; total serum protein: 5.1 g/dl; albumin: 0.9 g/dl; cholesterol: 302 mg/dl; renal and liver function tests were normal. Urine analysis revealed 4+ albumin with no casts or cells. Urinary protein excretion was 90 mg/m2 per hour. Blood and urine cultures were sterile; pericardial fluid analysis showed a protein level of 2.5 g/dl, sugar of 108 mg/dl, total cell count of 600 cells, 50% polymorphs, 50% lymphocytes, a cholesterol level of 130 mg/dl, and triglycerides of 356 mg/dl. The culture of the fluid was sterile	Hemoglobin: 8.8 g/dl; WBC: 9.1 × 109 /L; platelet count: 918 × 109/L; blood urea nitrogen: 30.5 mg/dL; creatinine: 1.2 mg/dL; total protein: 3.3 g/dL; albumin: 1.5 g/dL; total cholesterol: 560 mg/dL; triglyceride: 970 mg/dL; urinalysis protein: 3+; pericardial fluid analysis revealed WBC of 280/mm3, neutrophils 6%, lymphocytes 42%, monocytes 52%, lactate dehydrogenase of 100 IU/L, glucose of 95 mg/dl, protein of 600 mg/dL; pericardial fluid cytology tests and culture and blood cultures were all negative	Serum albumin: 1.8 g/dl; blood urea and serum creatinine were normal. Pericardial fluid showed no cells; protein of 150 mg/dl; glucose of 90 mg/dl; gram stain and PCR for tuberculosis were negative. The pericardial fluid protein: serum protein was 0.3; pericardial fluid LDH: serum LDH was 0.4. The pericardial fluid culture was sterile	Hemoglobin: 9.8 g/dl; WBC: 13.1 10e9/L; platelets: 630 × 109/L; creatinine: 69 umol/L, urea: 3.5; albumin level: 26 g/l; 24-hour urine protein: 3.4 g/day. Pericardial fluid cytology tests and culture were negative
Imaging	Chest X-ray showed cardiomegaly. Echocardiography revealed a massive pericardial effusion with cardiac tamponade. CT scan showed thrombosis and dilatation of the left brachiocephalic vein and superior vena cava with pericardial and pleural effusions	Chest X-ray revealed left pleural effusion and massive pericardial effusion. Echocardiography showed the collapse of the right atrium and ventricular	Chest X-ray showed massive cardiac enlargement. Echocardiography revealed pericardial tamponade with a right atrium and right ventricular collapse	Chest X-ray showed huge cardiomegaly. Echocardiography showed massive pericardial effusion
Outcome	Improved; drainage tubes were removed on the 15th day of hospitalization	Improved dramatically; the drainage tube was removed on the 12th day of pericardiocentesis	Improved clinically; echocardiogram repeated after one week later showed no effusion	Improved dramatically following pericardiocentesis; the drainage tube was removed on the 5th day of pericardiocentesis

Our patient was presented with nephrotic range proteinuria as well as other presentations of DDD. Pericarditis caused by b-hemolytic Streptococcus has been reported in pediatric patients and adults with post-streptococcal glomerulonephritis, both complicated by cardiac tamponade and managed by pericardiocentesis [18,19]. We suggest that this complication in our patient occurred because of hypoalbuminemia that is secondary to significant proteinuria. Other causes such as hypothyroidism, tuberculosis, and SLE were ruled out by appropriate investigations. Since the renal function tests at the time of occurrence of pericardial effusion were not very deteriorated, thus it is unlikely that pericardial effusion was a manifestation of uremia. Similarly, pericardial effusions due to viral infections (echovirus or coxsackievirus infection) were unlikely since the nature of the pericardial effusion was transudate. Multiple case reports indicate that minoxidil can cause large volume pericardial effusions [20] requiring discontinuation of this medication; our case was not on minoxidil. To the best of our knowledge, this is the first reported case of cardiac tamponade in DDD.

## Conclusions

With this report, we would like to remind clinicians that pericardial tamponade is a possible and uncommon but serious complication of DDD with nephrotic range proteinuria, which requires prompt and lifesaving pericardiocentesis. Patient edema was not correlated with the amount of pericardial effusion, which might be due to the slow accumulation of pericardial fluid. A detailed cardiac assessment should be carried out when a child with nephrotic syndrome or nephrotic range proteinuria presents with chest pain, tachycardia, tachypnea, dyspnea, and cardiomegaly.
